# Preparation and Characterization of Eel (*Anguilla*) Bone Collagen Based on Intelligent Algorithm

**DOI:** 10.3390/foods14244338

**Published:** 2025-12-16

**Authors:** Li Yuan, Jiayu Lu, Yingxi Jia, Zitao Guo, Ruichang Gao

**Affiliations:** School of Food and Biological Engineering, Jiangsu University, Zhenjiang 212013, China; yuanli24@163.com (L.Y.); ljy23412@163.com (J.L.); 2212318035@stmail.ujs.edu.cn (Y.J.)

**Keywords:** eel bones, collagen, extract, characterization, BP neural network, genetic algorithm

## Abstract

Eel (*Anguilla*) is an aquatic animal with high nutritional value and multiple health benefits for the human body. To fully utilize its processing by-products fish bone, this study optimized the enzymatic preparation process of using BP neural network and GA genetic algorithm, with collagen extraction yield as the key evaluation metric, and characterized the properties of the obtained collagen. The results demonstrated that the optimal extraction conditions for eel bone collagen were as follows: enzyme dosage of 2%, hydrolysis time of 2.65 h, solid-to-liquid ratio of 1:22, and ultrasonic pretreatment for 21 min at 250 W power, achieving an extraction yield of 57.6%. The main amino acids identified were glycine, glutamic acid, proline, and arginine. SDS-PAGE electrophoresis revealed that eel bone collagen exhibited structural characteristics of type I collagen. Raman spectroscopy and X-ray diffraction indicated an intact triple-helix structure with partial ordered features. The DSC and TGA results demonstrated good thermal stability, with a denaturation temperature of 106.73 °C. SEM imaging displayed a loose, porous fibrous network structure, while rheological analysis suggested potential biomedical material properties. The findings of this study provide fundamental data for the high-value utilization and development of eel bone resources.

## 1. Introduction

Collagen is a high molecular weight protein with important biological functions. Its unique physicochemical properties endow it with physiological activities such as preventing and treating osteoporosis, beautifying and anti-aging, promoting wound healing, preventing type II diabetes, and reducing fat intake [1]. Fish bones are rich in collagen and are a high-quality raw material for collagen extraction. Extraction methods for collagen can be classified into five categories based on the extraction medium: acid [2], alkali [3], enzyme [4], salt, and hot water extraction [5]. In recent years, composite extraction techniques combining physical means such as ultrasound or pressure assistance with the aforementioned traditional methods have gradually gained attention due to advantages like low energy consumption and high extraction yield [6].

Eel (*Anguilla*) is an aquatic animal with high nutritional value and various health benefits. It is rich in nutrients such as protein, unsaturated fatty acids, various vitamins, and minerals. Compared to other fish and meats, the nutritional value of eel is no less impressive. The high-quality protein and various essential amino acids it contains give it potential application value in the fields of health foods and medicine. In recent years, due to the rise in new production areas, eel output has increased annually. China’s eel farming output has shown a year-on-year growth trend over the past three years [7]. During eel processing, a large amount of by-products such as heads and bones are generated. However, most of these are discarded as waste, resulting in resource waste and even environmental pollution.

Fish by-products, such as skin, bones, and scales, are rich in collagen and thus hold potential economic value. Previous studies have successfully extracted collagen from various fish species, including tilapia [4], grass carp [8], and sturgeon [9], which can serve as alternatives to mammalian collagen. Despite the well-documented nutritional and medicinal benefits of eel [10] and the significant amount of by-products generated during eel processing, there is still a lack of comprehensive research on the structural and functional characteristics of collagen derived from eel. Research has found that eel bones contain a substantial amount of active components [11]. If utilized rationally, this could not only reduce resource waste but also achieve green and sustainable development in aquatic product processing. Therefore, in-depth research on eel bones is necessary to provide new ideas and references for their full utilization.

The BP neural network is a type of multi-layer feedforward network that uses the backpropagation algorithm to adjust network weights to minimize error. It is suitable for classification and regression tasks and can learn nonlinear relationships. The Genetic Algorithm (GA) is an optimization algorithm based on natural selection and genetic mechanisms. It performs global, multi-point search by simulating biological evolution, using probabilistic transitions rather than deterministic rules to find the optimal solution. Combining the local search capability of the BP neural network with the global search capability of the genetic algorithm can leverage the advantages of both, improving the training efficiency and performance of the neural network [12]. Traditional orthogonal experimental designs and response surface methodology (RSM), while widely employed in process optimization, are constrained by notable limitations. Orthogonal experiments often inadequately capture variable interactions and exhibit limited capacity for global optimization, whereas RSM models rely on idealized assumptions and demonstrate poor adaptability to dynamic conditions. However, the design of neural networks can effectively circumvent subjective errors and analytical biases caused by human factors in traditional information processing methods. This technology, directly based on experimental observation data and through its excellent adaptive learning mechanism and nonlinear transformation characteristics, autonomously constructs the network architecture, thereby ensuring the objectivity and reliability of the output results [13]. Furthermore, as a black-box model, neural networks exhibit strong environmental adaptability, excellent fault tolerance, and high simulation efficacy for complex unknown systems, enabling effective knowledge discovery in unknown domains and intelligent solving of complex problems [14]. With increasing consumer expectations for higher-quality aquatic products and growing demand for low-carbon processing practices, conventional experience-based optimization methods in fish processing have become inadequate for meeting the challenge of multi-objective synergistic regulation. In recent years, artificial intelligence technologies—particularly deep learning—have emerged as promising solutions for parameter prediction, quality control, and energy efficiency enhancement in fish processing, owing to their strong capacity for nonlinear modeling and adaptive optimization [15,16,17].

This study preprocessed the bones of artificially farmed Fujian American eel (*Anguilla*) to produce bone powder and determined their physicochemical indicators. Ultrasound-assisted enzymatic extraction was used to extract collagen from the fish bones, and the extraction yield of collagen from the crude enzymatic hydrolysate was used as the indicator for process optimization based on intelligent algorithms. Finally, various methods were employed to analyze the properties and evaluate the performance of the eel bone collagen. The experimental results are expected to provide new perspectives and a theoretical basis for the development and utilization of eel bone by-products, contributing to sustainable development.

## 2. Materials and Methods

### 2.1. Experimental Materials and Instruments

Fujian American eel bones, purchased from Fujian Fuqing Eel Road Trading Co., Ltd., Fujian, China.

NaOH, EDTA-2Na, isopropanol, n-propanol, glacial acetic acid, NaCl, HCl, anhydrous sodium acetate, citric acid monohydrate, p-dimethylaminobenzaldehyde, perchloric acid, etc., all of analytical grade, purchased from China National Pharmaceutical Group Chemical Reagent Co., Ltd. (Beijing, China); (Porcine) pepsin, Chloramine T, purchased from Shanghai Macklin Biochemical Co., Ltd. (Shanghai, China).

Grinder (Royalstar); PRACTUM124 analytical balance (Sartorius Scientific Instruments Co., Ltd., Göttingen, Germany); Freeze dryer (Boyikang Experimental Instrument Co., Ltd., Beijing, China); pHSJ-35 pH meter (Yidian Scientific Instrument Co., Ltd., Shanghai, China); Multifunctional tabletop centrifuge (Eppendorf AG, Hamburg, Germany); UV-1900i UV-Vis spectrophotometer (Rayleigh Analytical Instrument Corp, Beijing, China); Water bath (Jinyi Instrument Technology Co., Ltd., Changzhou, China); IKA-VORTEX 2 vortex mixer (IKA-Werke GmbH & Co. KG, Staufenberg, Germany); HYL-C2 combination shaker (Taicang Shiqiangwen Experimental Equipment Co., Ltd., Suzhou, China); Smart Lab multifunctional X-ray diffractometer (Rigaku Corporation, Tokyo, Japan); DSC-3 differential scanning calorimeter (Mettler-Toledo Ltd., Zurich, Switzerland); DXR laser micro-Raman spectrometer (Thermo Fisher Scientific Inc., Waltham, MA, USA); MIRA field emission scanning electron microscope (TESCAN ORSAY HOLDING, a.s., Brno, Czech Republic); DHR-1 rotational rheometer (Waters Corporation, Shanghai, China); WM-300GVDE/3 multi-frequency ultrasonic cleaning machine (Shanghai Weimi Technology Co., Ltd., Shanghai, China).

### 2.2. Preparation and Determination of Eel Bone Powder

#### 2.2.1. Preparation of Eel Bone Powder

Referencing the method of Bandeira et al. [18], thawed frozen eel bones were cut into segments less than 5 cm in length, and impurities such as internal organs were removed. The bones were repeatedly rinsed with clean water until free of blood. The bones were soaked in a 5% NaOH solution for 2 h to remove residual meat, with a sample-to-solution mass-to-volume ratio of 1:2 (*m*/*v*). After soaking, the bones were washed thoroughly with distilled water. The bones were then soaked in a 0.5 mol·L^−1^ ethylenediaminetetraacetic acid disodium salt (EDTA-2Na) solution for 72 h to remove minerals, with the solution changed every 12 h. After soaking, the bones were washed thoroughly with distilled water. The bones were treated with 10% isopropanol to remove excess lipids, with a solid-to-liquid ratio of 1:5 (*m*/*v*), and slowly stirred at 4 °C for 24 h for defatting, with the treatment solution changed every 12 h. After defatting, the bones were removed, rinsed 3–4 times with distilled water, drained, and stored at −40 °C. The bones were freeze-dried using a freeze dryer for 48 h, and then ground into powder using a grinder for later use.

#### 2.2.2. Determination of the Basic Components, Amino Acids and Calcium Content of Eel Bone Powder

Moisture determination is referred to as GB/T 5009.3-2016 [19]. Ash determination is referred to as GB/T 5009.4-2016 [20]. Crude protein determination is referred to as GB/T 5009.5-2016 [21]. Crude fat determination is referred to as GB/T 5009.6-2016 [22]. Amino acid composition determination is referred to as GB/T 5009.124-2016 [23]. The determination of calcium content shall be carried out by the method of inductively coupled plasma mass spectrometry (ICP-MS) in accordance with the national GB/T 5009.268-2016 [24].

### 2.3. Preparation of Eel Bone Collagen Protein

Referencing the method of Ahmed et al. [25], approximately 1.00 g of pretreated bone powder was weighed, 20 mL of ultrapure water was added, and mixed well. The pH was adjusted to approximately 2.0 using 0.5 mol/L glacial acetic acid. Ultrasonic treatment was applied (250 W, 20 min, 40 Khz). Pepsin (porcine origin) equivalent to 2% of the original bone powder mass was added. The mixture was incubated with shaking (37 °C, 2 h, 160 rpm). Enzyme inactivation was performed (water bath, 95 °C, 10 min). Centrifugation was carried out (4 °C, 10,000× *g*, 15 min). The supernatant was collected as the crude collagen extract.

Referencing the method of Tamilmozhi et al. [26], NaCl solid powder was slowly added to the crude extract to a final concentration of 0.9 mol/L, controlling the addition rate to ensure complete dissolution and no solid residue. The treated solution was left to stand (4 °C, 12 h), followed by centrifugation (4 °C, 10,000× *g*, 30 min) to obtain the salt-precipitated pellet. The precipitate was collected and redissolved in 0.5 mol/L glacial acetic acid. Gradient dialysis was performed sequentially using 0.1 mol/L glacial acetic acid and ultrapure water until no chloride ions were detected in the solution. The sample was freeze-dried to obtain collagen, which was stored in a −80 °C freezer.

### 2.4. Calculation of the Extraction Rate of Eel Bone Collagen

#### 2.4.1. The Drawing of the Standard Curve of Hydroxyproline

Precisely 10.0 mg (accurate to 0.1 mg) of hydroxyproline standard was weighed into a beaker, dissolved in pure water, quantitatively transferred to a 10 mL brown volumetric flask, and 1 drop of 6 mol/L hydrochloric acid solution (approx. 50 μL) was added to prepare the hydroxyproline standard stock solution. Transfer 0.25 mL of the stock solution to a 25 mL volumetric flask and diluted to the mark with pure water to prepare a 5.0 μg/mL standard working solution. Serial dilution with pure water yielded standard working solutions of 0.5, 1.0, 1.5, and 2.0 μg/mL [27].

The hydroxyproline standard working solution (4.0 mL) was pipetted into a test tube, and 2.0 mL of Chloramine T solution was added. After mixing, it was left at room temperature for 20 min. Then, 2.0 mL of color developing agent was added, vortex mixed, and the reaction proceeded in a water bath (60 °C, 20 min). The test tubes were removed, cooled under running water for 3–5 min, and then left at room temperature for 30 min. Using deionized water as the blank control, the absorbance was measured at 558 nm, with the average of three parallel measurements taken. A linear regression equation was fitted with the hydroxyproline standard solution concentration (μg/mL) as the abscissa (X) and the absorbance after blank subtraction as the ordinate (Y), and the correlation coefficient (R^2^) was calculated.

#### 2.4.2. Determination of Hydroxyproline Content and Extraction Rate

For the crude collagen extract obtained in Section 2.3, precisely 1 mL of the sample was transferred to a hydrolysis tube, avoiding wall adhesion. Add 20 mL of 6 mol/L hydrochloric acid solution, sealed, and placed in a 105 °C drying oven for hydrolysis for 20 h. The hydrolysate was completely transferred to a 100 mL volumetric flask. The hydrolysis tube was rinsed sequentially with 10 mL of 6 mol/L hydrochloric acid and 10 mL of pure water, and the rinsates were combined into the volumetric flask, which was then diluted to the mark with pure water. Take 2 mL of the above diluted solution, and 8 mL of pure water was added for a 5-fold dilution, ensuring the hydroxyproline concentration fell within the range of 0.5–5.0 μg/mL. Take 4.0 mL of the diluted solution, and the absorbance was measured according to the standard curve method (same as Section 2.4.1). The concentration of hydroxyproline in the test solution was calculated using the obtained linear regression equation [27]. The calculation formula is as follows:

Hydroxyproline content (%) = (c × 100 × n)/(m × 1000) × 100%,(1)

where: c is the hydroxyproline concentration in the test solution obtained from the standard curve, in μg/mL; m is the sample mass, in mg; 100 is the volume of the volumetric flask to which the hydrolysate was diluted, in mL; n is the dilution factor of the hydrolysate in the stoppered test tube; 1000 is the conversion factor.

Collagen extraction rate (%) = (X × m × F × V)/840,(2)

where: X is the hydroxyproline content obtained from Formula (1); F is the collagen conversion factor, value 11.1; m is the sample mass in (1), in mg; V is the volume of the crude collagen extract, in mL; 840 is the theoretical collagen value.

### 2.5. Optimization of Eel Bone Collagen Extraction Process

In this step, a BP neural network was used to optimize the eel bone collagen extraction process. and five key process factors were selected: enzymatic hydrolysis time, enzyme addition amount, solid-to-liquid ratio, ultrasonic pretreatment time, and ultrasonic power. The factor levels for the orthogonal experimental design are shown in Table 1. The collagen extraction yield calculated in Section 2.4.2 was used as the evaluation criterion for process optimization.

#### 2.5.1. BP Neural Network Design

Matlab 2016b software was used for programming. A network structure design including input layer, hidden layer, and output layer was established. The input layer was set to 5 neurons: enzymatic hydrolysis time, enzyme addition amount, solid-to-liquid ratio, ultrasonic pretreatment time, and ultrasonic power. GA was used to determine the optimal number of hidden layer nodes as 15 neurons to improve the model’s predictive performance [28]. The extraction yield was set as the output layer. Therefore, the network topology was of the 5-15-1 type. This network was used for simulation to analyze the influence of each factor on the collagen extraction yield.

For sample selection, a portion of all random samples was used as training samples, and another portion was used as test data for network performance [29]. This study obtained 32 groups of data samples by combining orthogonal and random experiments. Select 28 groups as training samples, and the remaining 4 groups were used as test samples. Each group of values in the samples included the input vector and the expected output vector for the neural network. During network training, referencing the method of Chen et al. [30], the training target error, learning rate, and maximum training steps were set. After setting the network structure (number of network layers, number of neurons per layer, and neuron activation functions), learning method, and target error, the network was run.

#### 2.5.2. GA Genetic Algorithm Optimization

GA considered a complete experimental operation as an individual (its attributes represented by an array of the corresponding process parameter values), and the output vector extraction yield of the experiment was regarded as the fitness value of an individual. Referencing the method of Chen et al. [30], the relevant parameters for GA were set.

### 2.6. Characterization of Eel Bone Collagen Protein

#### 2.6.1. Amino Acid Composition Analysis

Amino acid composition determination is referred to as GB/T 5009.124-2016 [23].

#### 2.6.2. SDS-PAGE

Referencing the method of Sun et al. [31], a certain mass of freeze-dried collagen sample was dissolved in a 5% SDS solution to prepare a protein solution of appropriate concentration. It was heated (85 °C, 1 h) to fully dissolve the protein. After cooling, it was centrifuged (25 °C, 10,000 rpm, 15 min) to obtain the supernatant. The supernatant was mixed with loading buffer in a certain volume ratio, boiled for 5 min, and cooled. Take 10 μL of sample. Using a 4% stacking gel and a 10% separation gel, electrophoresis was stopped when the dye front migrated to approximately 3–5 mm from the bottom of the gel. The gel was then immersed in Coomassie Brilliant Blue R-250 staining solution and gently agitated on a shaker for 30 min. After discarding the staining solution, destaining solution was added, and the gel was agitated until the background became transparent. The destained gel was placed in ultrapure water and stored at 4 °C.

#### 2.6.3. X-Ray Diffraction Analysis (XRD)

A multifunctional X-ray diffractometer was used for XRD analysis of eel bone collagen. An appropriate amount of freeze-dried collagen was placed in the center of the sample holder. Cu Kα radiation was used, λ = 0.154 nm, scanning speed 5°/min, scanning range 10~80°. The X-ray diffraction pattern was recorded.

#### 2.6.4. Heat Distortion Temperature Determination

Approximately 8 mg of freeze-dried sample was weighed into a DSC crucible, the temperature program was set (temperature range 30~140 °C, heating rate 10 °C∙min^−1^), nitrogen flow rate 50 mL∙min^−1^.

#### 2.6.5. Raman Spectroscopy Analysis

Scanning was performed using a diode laser with an excitation wavelength of 532 nm and power of 10 mW. The instrument resolution was 1 cm^−1^, and the spectrum was obtained in the measurement range of 500~3500 cm^−1^.

#### 2.6.6. Thermogravimetric Analysis (TGA)

Briefly, 5 mg of sample was weighed, and the initial test temperature was 30 °C, heating rate 10 °C∙min^−1^, temperature range 30~800 °C, carrier gas nitrogen, flow rate 20 mL∙min^−1^.

#### 2.6.7. Scanning Electron Microscope Analysis (SEM)

The surface microstructure of the collagen was observed at magnifications of 50× and 1000× under a 5.0 kV accelerating voltage, and scanning images were acquired.

#### 2.6.8. Rheological Analysis

Referencing the method of Li et al. [32], an appropriate amount of freeze-dried collagen sample was dissolved in phosphate-buffered solution, fully mixed to prepare a 10 mg/mL collagen solution. Using a 40 mm, 1° cone plate, the frequency sweep range was set to 0.1~10 Hz, strain rate 30%. The variation in the storage modulus (elastic modulus) G′ and loss modulus (viscous modulus) G″ of the collagen solution with oscillatory frequency was studied.

### 2.7. Data Statistics and Analysis

SPSS 26 software was used for analysis of variance (ANOVA), with *p* < 0.05 considered statistically significant. Origin 2022 software was used for plotting. Matlab 2016b software was used for training and optimizing the neural network model.

## 3. Results and Discussions

### 3.1. Basic Components and Calcium Content

The basic components and calcium content of eel bone powder are shown in Table 2. The highest component content in eel bone powder is crude protein. This is because the fish bones had already undergone pretreatment and freeze-drying, removing a large amount of water, fat, and minerals, resulting in lower amounts of other components. These results indicate that appropriate pretreatment of eel bone powder before collagen extraction can effectively remove some impurities, increase protein purity, and avoid impacts on the subsequent collagen extraction process and product processing. Among the measured results, the crude protein content of eel bones was the highest (75.15%), higher than that of cod bones (35.78%) and salmon bones (48%), but lower than that of snapper bones and grouper bones (78.5–82.36%) [33,34,35]. Typically, excessively high fat content can lead to off-flavors during the enzymatic hydrolysis of raw materials and affect their shelf life. However, the fat content of eel bone powder is only 4.59%, classifying it as a low-fat raw material, significantly reducing potential food safety issues. Therefore, the prepared eel bone powder has the advantages of high protein and low fat, and may be a high-quality raw material for preparing natural antioxidant peptides [36].

Because EDTA-2Na was used in the preparation of the bone powder to chelate most of the free calcium in the fish bones, the residual 0.116 mg/g is likely mainly bound calcium (structural calcium), such as hydroxyapatite crystals (Ca_10_(PO_4_)_6_(OH)_2_) [37] or collagen-bound calcium [38]. The former is the main form of calcium in fish bones and is difficult to remove completely with EDTA, while the latter is calcium bound to carboxyl groups/phosphorylation sites on collagen fibers, accounting for about 5–10%. Compared to the original total calcium content in fish bones (typically 30–100 mg/g), the measured calcium content in the bone powder accounts for only 0.1–0.4%, indicating that EDTA treatment has removed the vast majority of calcium.

### 3.2. Amino Acid Composition

The amino acid composition of eel bone powder is shown in Table 3. A total of 16 amino acids were detected, including 7 essential amino acids. The contents of Glycine (22.44%), Proline (11.95%), Hydroxyproline (10.77%), and Arginine (9.72%) were relatively high. Research by Nilesh et al. [39]. indicated that antioxidant peptides contain a relatively high proportion of hydrophobic amino acids. The proportion of hydrophobic amino acids in eel bone powder reached 52.50%, suggesting that the protein in eel bone powder may possess certain antioxidant effects after hydrolysis.

### 3.3. Optimization of Eel Bone Collagen Extraction Process

#### 3.3.1. Test Data and Training Neural Network

Table 4 shows the experimental values under different parameter combinations, with the extraction yield in the last column as the output layer. During model establishment, the model first predicts and trains on the 28 sets of parameter combinations from the experiment, and continuously adjusts the model parameters based on the validation sets until the model’s accuracy meets the requirements. Through repeated self-learning and training of the neural network, the optimal neural network model parameters were obtained, and the training was concluded.

**Figure 1:**
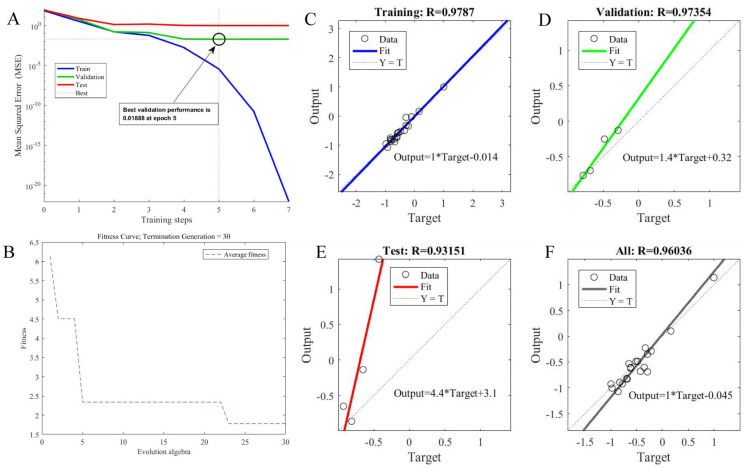
(**A**)—error drop curve of neural network; (**B**)—the fitness curve of the extraction rate; the regression performance of extraction rate: (**C**)—training curve; (**D**)—validation curve; (**E**)—test curve; (**F**)—Comprehensive curve.

As shown in Figure 1A, the training process of the neural network was monitored by the Mean Square Error (MSE). As a common indicator for measuring model prediction accuracy, MSE can intuitively reflect the optimization process of network performance; a smaller MSE value indicates higher model accuracy [40]. During the neural network training process, the network error curve gradually approached the target error value of 0.0001 as the training steps increased. At Section 5, the model met the expected performance requirements: the MSE of the validation set dropped to 0.018888, which was the global optimum; the test set error converged synchronously, indicating good generalization ability of the model; the subsequent training process remained stable without overfitting. This optimization trajectory indicates that the network architecture design is reasonable, capable of effectively learning data features, and the training parameter settings are appropriate, ensuring rapid convergence; the model achieved ideal accuracy in a relatively short time, demonstrating excellent computational efficiency.

#### 3.3.2. Regression Performance and Optimization Results

The regression performance of the model determines the accuracy of the prediction results. As shown in Figure 1C–F, the model exhibited good regression performance with a high fitting accuracy rate. The GA-BP neural network model achieved fitting accuracy rates of 97.87%, 97.354%, 93.151%, and 96.036% for the training, validation, test, and all datasets, respectively. This indicates that the neural network model has excellent explanatory ability. The error difference between the training set and validation set was only 0.516%, the simulation error was small, the precision was high, ensuring good simulation results. The model successfully established a high-precision mapping relationship between the five input parameters and the target output, with good prediction stability, strong generalization ability, and high practical value, meeting engineering accuracy requirements. The model performance can be further improved by increasing the training sample size, optimizing network depth, etc.

Perfecting the fitness of the optimized parameters of the network model can effectively improve the performance and convergence rate of the GA-BP neural network model. After setting the basic parameters of the GA, the fitness function was used for debugging, and optimization was performed with the genetic algorithm aiming for maximum extraction yield. After multiple iterations analyzing the optimal and average weight parameters, the maximum fitness value could be obtained. By adjusting the enzymatic hydrolysis time, enzyme addition amount, solid-to-liquid ratio, ultrasonic treatment time, and ultrasonic power, the highest and lowest extraction yields were obtained. As shown in Figure 1B, after 30 iterations, the optimized result was: the optimal extraction yield was 56.88%, and the optimal process parameters were enzymatic hydrolysis time 2.6536 h, enzyme addition amount 2.0194%, solid-to-liquid ratio 1:21.8036, ultrasonic treatment time 21.0438 min, ultrasonic power 248.5012 W.

A verification experiment was conducted according to the process parameters corresponding to the optimization results (taking approximate values), and the results are shown in Table 5.

From the data in Table 5, the extraction yield of the verification experiment was 57.6%, which is 0.72% higher than the model optimization value, with a relative error within ±0.02%, meeting the accuracy requirements of the BP neural network model. This indicates that optimizing the eel bone collagen extraction process using this neural network combined with the genetic algorithm can achieve a relatively ideal extraction yield value. Therefore, the optimal process optimized by the GA-BP network is: enzymatic hydrolysis time 2.65 h, enzyme addition amount 2%, solid-to-liquid ratio 1:22, ultrasonic treatment time 21 min, ultrasonic power 250 W. The extraction rate under this condition was higher than that of acid soluble and enzyme soluble eel skin and bone collagen reported by Zhang et al. [11].

### 3.4. Characterization of Eel Bone Collagen (EBC)

#### 3.4.1. Amino Acid Composition Analysis of EBC

The amino acid composition of eel bone collagen is shown in Table 6. The contents of Glycine (24.44%), Hydroxyproline (21.17%), Glutamic acid (13.24%), and Proline (12.88%) were relatively high. The proportion of essential amino acids in eel bone collagen was 23.80%, lower than the recommended ratio for high-quality protein in the FAO/WHO ideal pattern (EAA/TAA: 40%), indicating that eel bone protein is not suitable as a daily dietary protein. Peptides with high antioxidant activity generally contain a high proportion of hydrophobic amino acids [39]. The proportion of hydrophobic amino acids in eel bone collagen reached 60.93%, slightly lower than that of eel skin (61.79%) [41]. Therefore, it is speculated that its enzymatic hydrolysate may contain highly active antioxidant peptides.

#### 3.4.2. Molecular Weight Analysis by SDS-PAGE of EBC

**Figure 2:**
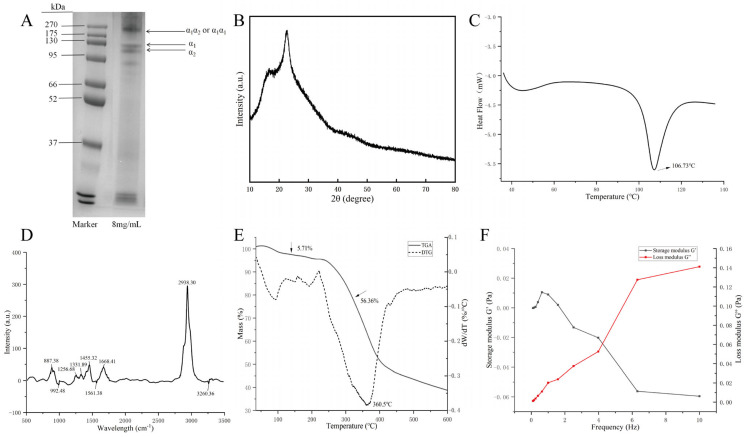
(**A**)—electrophoretic pattern of eel bone collagen; (**B**)—XRD plot of eel bone collagen; (**C**)—DSC curve of eel bone collagen; (**D**)—Raman spectrum of eel bone collagen; (**E**)—TGA and DTG curves of eel bone collagen; (**F**)—rheological characteristics of eel bone collagen solution.

The electrophoretogram of eel bone collagen (Figure 2A) is similar to those of crocodile bone collagen (type I) [42], mullet bone collagen (type I) [43], and acid-soluble collagen from bighead carp bone (type I) [31], suggesting that it belongs to type I collagen and has a relatively intact triple-helical structure. There are two clear bands near 130 kD, presumed to be the α_1_ and α_2_ chains of collagen [44]; a clear band near 270 kD is presumed to be the α_1_ and α_2_ chain complex (α_1_α_2_ or α_1_α_1_) [45]; faint bands above 270 kD may be intact collagen molecules and other complexes [46]; bands below 130 kD may be due to degradation by pepsin during extraction, as its specific cleavage might cause partial hydrolysis of collagen, producing low molecular weight fragments.

#### 3.4.3. X-Ray Diffraction Analysis (XRD) of EBC

X-ray diffraction technology can be used to analyze the crystal structure of materials, resolving changes in molecular arrangement, conformation, and crystal morphology. The X-ray diffraction pattern of eel bone collagen is shown in Figure 2B. A significant diffuse diffraction peak was observed near 2θ = 22°, primarily originating from the diffuse scattering caused by the numerous complex structural hierarchies within the collagen molecules, reflecting the lateral distance between collagen molecular chains, related to the close packing of the triple-helical structure, representing the amorphous dispersed structure of type I collagen [46]. Around 2θ = 16°, there is a weaker and broader diffuse peak, belonging to the secondary diffraction peak of type I collagen, reflecting another lateral arrangement mode of collagen molecular chains. The diffraction peaks after 30° are small and sharp, reflecting the distance between adjacent amino acid residues on the triple-helical structure of collagen [47]. This indicates that eel bone collagen contains both ordered crystalline regions, such as the triple-helical structure and the ordered arrangement of collagen fibers [48], and amorphous regions, such as telopeptide regions and interfibrillar connection regions [49]. Its overall structure lies between completely crystalline and completely amorphous, belonging to a mesocrystalline structure or partially ordered structure. This partially ordered structure allows collagen to provide both mechanical strength and maintain a certain flexibility [50], adapting to the functional requirements of biological tissues and meeting the criteria for biomaterials.

#### 3.4.4. Differential Scanning Calorimetry (DSC) Thermograms Analysis of EBC

Thermal stability is a key indicator reflecting a material’s ability to maintain performance in high-temperature environments and is one of the important indicators reflecting the physicochemical properties of collagen. Thermal analysis of the sample was performed using a differential scanning calorimeter, and the results are shown in Figure 2C. As the temperature increased, a downward endothermic peak appeared in the DSC curve, indicating the thermal denaturation temperature (Td) of the sample was 106.73 °C. At this point, hydrogen bonds in the hydration network surrounding the collagen molecules break, free water is released, intermolecular cross-links decrease, leading to the unwinding of its triple-helical structure, and the conformation changes from ordered to random, accompanied by mass loss and changes in physicochemical properties [11].

Many factors affect the thermal stability of collagen, including the source of the raw material, season, amino acid composition, and sequence [51]. Some studies have shown that collagen from internal sources in fish has better thermal stability than that from external sources [52]; research by Zhang et al. [11] showed that the thermal denaturation temperature of eel skin collagen was 74.34 °C, significantly lower than that of eel bone collagen (106.73 °C). Secondly, the same protein from different species of fish has different thermal denaturation temperatures; for bone collagen, the thermal denaturation temperature of eel bone collagen prepared in this study was significantly higher than that of bighead carp (31.22 °C) [31], crocodile (38.2 °C) [42], and mullet (32 °C) [43]. The content of amino acids (proline and hydroxyproline) in collagen is an important factor affecting the thermal denaturation temperature; the higher their content, the higher the thermal denaturation temperature [53]. The above results indicate that eel bone collagen has good thermal stability, certain safety, and performance stability, and can be widely used in energy and chemical industries, medical and environmental protection, and food processing, possessing good application potential.

Furthermore, the regular crystalline structure of proteins usually corresponds to a higher thermal denaturation temperature [54]. Therefore, the DSC results, to some extent, reflect the integrity of the molecular structure and the characteristics of the crystal structure of eel bone collagen, which is consistent with the XRD results. The clarity of certain characteristic peaks in the XRD pattern directly reflects the regularity of the triple-helical structure; the more ordered the structure, the higher its thermal stability, requiring a higher temperature to unwind; as well as some long-period diffraction peaks reflecting the tightness of the intermolecular arrangement of collagen fibers; tightly arranged fibers, through more intermolecular cross-links (such as hydroxylysine-derived covalent bonds), significantly enhance thermal stability [54].

#### 3.4.5. Raman Spectroscopy Analysis of EBC

Raman spectroscopy is an analytical technique based on the Raman scattering effect. Compared to infrared spectroscopy, it has a wider range of applications, is not interfered with by water, enables fast real-time analysis and high spatial resolution, and does not cause damage or consumption to the sample. As shown in Figure 2D, characteristic absorption peaks of collagen exist in the Raman spectrum of eel bone collagen, including amide I, amide II, amide III, amide A, and amide B bands. The amide A band has an absorption peak at 3260.36 cm^−1^, mainly caused by N-H stretching vibration. Its peak position is sensitive to hydrogen bond strength and can form complexes with hydrogen bonding. The amide B band has an absorption peak at 2938.30 cm^−1^, mainly originating from the coupling of C-N stretching vibration and CH_2_ vibration. Its intensity is usually weak and adjacent to the amide A band.

The amide I, II, and III bands serve as absorption peaks for the protein peptide backbone structure. Their positions, shapes, and intensities directly reflect the integrity of the triple-helical structure and molecular conformation characteristics of collagen molecules. The absorption peak of the amide I band is located near 1668.41 cm^−1^, caused by C=O (carbonyl) stretching vibration, and is very sensitive to the secondary structure of proteins, making it a characteristic absorption peak for the secondary structure of collagen molecules [55]. The absorption peaks of the amide II and amide III bands appear at 1561.38 cm^−1^ and 1256.68 cm^−1^, respectively, mainly caused by C-N stretching vibration and N-H bending vibration [55]. The amide II band is less sensitive to protein secondary structure than the amide I band and is often used as a supplement for analyzing protein secondary structure. The amide III band can reflect the integrity of the collagen triple-helical structure and is related to the vibration of the -CH_2_- groups of glycine and proline residues [52]. The structural information of collagen reflected by this Raman spectrum is similar to that of type I collagen extracted from fish bones by Asaduzzaman et al. [56].

#### 3.4.6. Thermogravimetric (TGA) and Derivative Thermogravimetry (DTG) Analysis of EBC

TGA can reflect the thermal stability of a material by measuring the mass change with temperature under a controlled temperature program, analyzing physical and chemical processes such as decomposition, adsorption, desorption, oxidation, and reduction during this process. The TGA and DTG curves of eel bone collagen are shown in Figure 2E. The TGA curve shows two mass loss stages: The first stage starts around 30 °C, with a mass loss of 5.71%, attributed to the removal of physically absorbed water from the collagen [57]. The second stage starts around 230 °C and completes around 600 °C, with a mass loss of 56.36%. This is the main stage of collagen thermal degradation [58]; the triple-helical structure of collagen unwinds at high temperature, peptide bonds break generating small peptides and free amino acids, and these small molecules are volatile, leading to mass loss. Additionally, amino acid side chains or peptide chains may undergo cyclization or oxidation reactions, generating low molecular weight compounds, further affecting mass. After the thermal degradation stage reaction ends, the TGA curve stabilizes, indicating that the residue (inorganic salts and carbonization products) can no longer undergo further thermal degradation, accounting for about 40% of the total mass. The above degradation behavior is consistent with typical type I collagen. The DTG curve shows the maximum mass loss rate (Tm) during the pyrolysis of eel bone collagen is at 360.5 °C, corresponding to the TGA curve.

#### 3.4.7. Rheological Analysis of EBC

The storage modulus (G′) represents the ability of collagen molecules to deform under external force, which can characterize the elastic response ability of the collagen network structure and reflect the reversible resistance of molecular chains to external deformation; the loss modulus (G″) represents the energy loss caused by the stretching of molecular chains within or between molecules under action of external force, which is related to the friction between molecules and structural reorganization. The viscoelastic curve of the eel bone collagen solution is shown in Figure 2F. When the oscillation frequency is in the range of 0.1 to 10 Hz, G′ gradually decreases and G″ gradually increases with the increase in the frequency, and G″ is always greater than G′, indicating that the collagen solution mainly shows viscous behavior, which is similar to the characteristics of collagen solution of red stingray at low concentration in the study of Chen et al. [59]. In addition, proline and hydroxyproline are key components of the protein triple helix structure and are essential for the sol-gel transition, and the content of proline and hydroxyproline in collagen molecules will also affect the gelation [60]. G″ is always greater than′, indicating that the sample is difficult to gel, which may be due to the fact that the sample solution concentration is too low. The frequency-weakening characteristics of G indicate that the elastic response of molecular chains has a time-dependent property and that the network structure dissociates partially under high frequency; the frequency-strengthening characteristics of″ indicate that the segmental motion of molecular chains lags behind the strain rate and that the reorganization of structural units requires energy dissipation. The above results show that the rheological of this eel bone collagen solution conforms to the characteristics of a weakly physically cross-linked gel and may be more suitable for making injectable biomaterials.

#### 3.4.8. Observation of EBC by SEM

**Figure 3:**
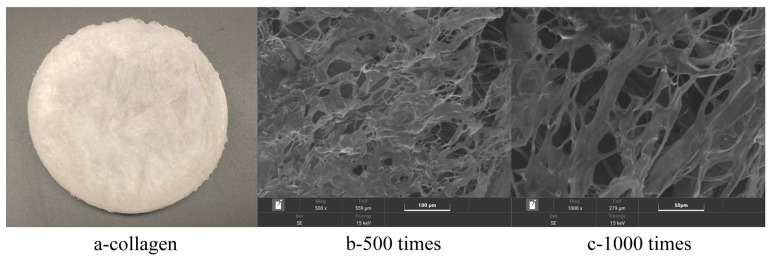
(**a**)—visually observed eel bone collagen protein; (**b**)—microstructure of eel bone collagen protein at 500 times; (**c**)—microstructure of eel bone collagen protein at 1000 times.

The freeze-dried eel bone collagen (Figure 3a) appears milky white, spongy, with uniform texture and softness, possessing recoverable elasticity. Under SEM (Figure 3b,c), it is observed that it exhibits a dense, multilayer aggregated network structure relatively uniform cellular porosity, which is consistent with the results of Zhang et al. [11]; the surface pore size of the sample is large, and the fiber surface is slightly rough, possibly caused by extensive water evaporation during freeze-drying; fragmented areas appear in some regions, which may be due to the action of pepsin. The porosity, fiber shape, and controllable biodegradability of collagen are key parameters for evaluating biomaterials. The fibers of eel bone collagen are relatively uniform, and the network structure tends to be consistent, potentially possessing good performance for biomedical and pharmaceutical applications [8]. It can provide a reference for the application in fields such as wound repair dressing, hemostatic agent, drug sustained-release carrier or matrix for cell proliferation.

## 4. Conclusions

This study successfully prepared eel bone collagen using ultrasound-assisted enzymatic extraction and optimized the extraction process using a GA-BP neural network. The optimal process parameters were: enzymatic hydrolysis time 2.65 h, enzyme addition amount 2%, solid-to-liquid ratio 1:22, ultrasonic treatment time 21 min, and ultrasonic power 250 W. Under these conditions, the extraction yield of eel bone collagen reached 57.6%.

The extracted collagen is characterized by a triple-helical structure, consistent with the structural features of type I collagen. It has a thermal denaturation temperature of 106.73 °C and exhibits good thermal stability. The collagen presents a loose, porous, and multi-layer aggregated fibrous network structure, aligning with the characteristics of a weakly physically cross-linked gel, indicating its potential as a biomedical material.

In summary, eel bones can serve as an ideal source of collagen. Through systematic development and research, they hold potential for applications in food, biomedicine, health products, and other fields. The findings of this study provide data support for the comprehensive utilization and development of fish bones, a major by-product of eel processing.

## Figures and Tables

**Table 1:** Factors of orthogonal test.

	Enzyme Hydrolysis Time/h	Amount of Enzyme Added/%	Slurry Ratio (g:mL)	Ultrasonic Pretreatment Time/min	Ultrasonic Power/W
1	1	1	20	10	125
2	2	1.5	25	15	250
3	3	2	30	20	375
4	4	2.5	35	25	500

**Table 2:** The basic composition of eel bone powder.

Basic Composition	Content
Moisture	5.85 ± 0.67%
Ash	1.79 ± 1.06%
Crude protein	75.15 ± 0.95%
Crude fat	4.59 ± 0.62%
Calcium	0.116 ± 0.08 mg/g

**Table 3:** The amino acid composition of eel bone powder.

Amino Acid	Content/%
Asp	5.47 ± 0.63
Thr	2.68 ± 1.57
Ser	3.74 ± 0.52
Glu	9.26 ± 1.80
Gly	22.44 ± 0.60
Ala	7.96 ± 0.75
Val	1.89 ± 1.22
Met	1.85 ± 1.21
Ile	1.30 ± 0.84
Leu	2.82 ± 0.99
Tyr	0.91 ± 0.69
Phe	2.29 ± 1.57
His	2.83 ± 2.16
Lys	3.94 ± 0.72
Arg	9.72 ± 0.97
Pro	11.95 ± 1.08
Hyp	10.77 ± 1.15

**Table 4:** Experimental data.

Samples	Enzyme Hydrolysis Time/h	Amount of Enzyme Added/%	Slurry Ratio/(g:mL)	Ultrasonic Pretreatment Time/min	Ultrasonic Power/W	Extraction Rate/%
1	2	1.5	25	15	125	37.34 ± 3.92
2	2	2.5	30	10	375	33.30 ± 0.87
3	1	1.5	30	25	250	30.10 ± 2.81
4	3	2	30	20	125	33.82 ± 1.23
5	1	1	20	10	125	28.05 ± 2.10
6	1	2.5	25	20	500	31.21 ± 0.67
7	3	1	25	25	375	29.80 ± 2.34
8	1	2	35	15	375	36.23 ± 0.98
9	4	2	25	10	250	44.32 ± 4.56
10	2	2	20	25	500	28.71 ± 1.09
11	3	2.5	20	15	250	52.78 ± 3.26
12	2	1	35	20	250	35.78 ± 4.21
13	4	1	30	15	500	33.15 ± 1.09
14	3	1.5	35	10	500	30.80 ± 2.01
15	4	2.5	35	25	125	37.07 ± 3.21
16	4	1.5	20	20	375	49.59 ± 4.50
17	1	1.5	30	25	125	29.05 ± 1.04
18	1	2.5	30	20	500	34.68 ± 1.12
19	1	2	35	15	125	29.00 ± 3.45
20	1	2	35	10	375	24.32 ± 2.34
21	2	2.5	30	10	125	38.30 ± 0.89
22	2	2	20	20	250	51.87 ± 5.60
23	2	1	35	20	500	37.60 ± 3.45
24	2	1.5	25	15	250	50.10 ± 2.30
25	3	1	35	10	500	31.44 ± 2.12
26	3	1.5	25	25	375	31.92 ± 4.50
27	3	2	25	20	375	35.24 ± 4.25
28	3	2.5	20	25	125	37.38 ± 3.42
29	4	2.5	30	15	125	33.31 ± 5.64
30	4	1.5	25	20	375	40.10 ± 4.28
31	4	2	20	25	250	53.84 ± 2.00
32	4	1	25	20	500	28.40 ± 3.56

**Table 5:** The optimization results and comparative validation.

Process Parameters	Parameter Value	Metric Type	Extraction Rate/%	Relative Error/%
Enzyme hydrolysis time	2.65 h	Model optimization value	56.88	——
Amount of enzyme added	2%			
Slurry ratio	1 g:22 mL	Test value	57.6	0.02
Ultrasonic pretreatment time	21 min			
Ultrasonic power	250 W			

**Table 6:** The amino acid composition of EBC.

Amino Acid	Content/%
Asp	7.90 ± 0.60
Thr	3.66 ± 1.04
Ser	4.57 ± 0.98
Glu	13.24 ± 1.00
Gly	24.44 ± 0.99
Ala	9.49 ± 0.32
Val	2.93 ± 0.03
Met	0.74 ± 0.56
Ile	2.40 ± 0.82
Leu	4.94 ± 1.02
Tyr	1.32 ± 0.98
Phe	3.11 ± 0.76
His	4.30 ± 1.21
Lys	6.02 ± 0.23
Arg	10.95 ± 0.34
Pro	12.88 ± 0.81
Hyp	21.17 ± 1.12

## Data Availability

The original contributions presented in this study are included in this article. Further inquiries can be directed to the corresponding authors.
